# A Novel Combination of High-Load Omega-3 Lysine Complex (AvailOm^®^) and Anthocyanins Exerts Beneficial Cardiovascular Effects

**DOI:** 10.3390/antiox11050896

**Published:** 2022-04-30

**Authors:** Paola Di Pietro, Rosario Lizio, Carmine Izzo, Valeria Visco, Antonio Damato, Eleonora Venturini, Massimiliano De Lucia, Gennaro Galasso, Serena Migliarino, Barbara Rasile, Michele Ciccarelli, Carmine Vecchione, Albino Carrizzo

**Affiliations:** 1 Department of Medicine, Surgery and Dentistry ‘‘Scuola Medica Salernitana’’, University of Salerno, 84081 Baronissi, Italy; pdipietro@unisa.it (P.D.P.); c.izzo26@studenti.unisa.it (C.I.); vvisco@unisa.it (V.V.); ggalasso@unisa.it (G.G.); serena.migliarino@studenti.unicz.it (S.M.); brasile@unisa.it (B.R.); mciccarelli@unisa.it (M.C.); cvecchione@unisa.it (C.V.); 2 Evonik Operations GmbH, 63457 Hanau, Germany; rosario.lizio@evonik.com; 3 Vascular Physiopathology Unit, IRCCS Neuromed, 86077 Pozzilli, Italy; antonio.damato@neuromed.it (A.D.); eleonora.venturini94@libero.it (E.V.); massidelucia.m@libero.it (M.D.L.)

**Keywords:** omega-3 lysine complex, anthocyanins, cardiovascular, oxidative stress

## Abstract

Omega-3 fatty acids have been shown to exert several beneficial effects in the prevention of cardiovascular and cerebrovascular diseases. The objective of the present study was to analyze the effects of a novel high-load omega-3 lysine complex, AvailOm^®^, its related constituents and a novel mixture of AvailOm^®^ with specific vasoactive anthocyanins on vascular function in mice resistance artery. Pressure myograph was used to perform vascular reactivity studies. Nitric oxide and oxidative stress were assessed by difluorofluorescein diacetate and dihydroethidium, respectively. Increasing doses of AvailOm^®^ exerted a dose-response vasorelaxation via AMPK-eNOS-mediated signaling. Omega-3 Ethyl Ester was identified as the main bioactive derivative of AvailOm^®^, being capable of inducing vasorelaxant action to the same extent of entire product. The combination of AvailOm^®^ with a mix of potent vasoactive anthocyanins (C3-glu + DP3-glu + Mal3-glu + Mal3-gal + PEO3-gal), strongly protected mesenteric arteries from vascular dysfunction and oxidative stress evoked by oxidized-LDL. These data demonstrate for the first time the direct effects of AvailOm^®^ on resistance arteries. The evidence that the combination of specific vasoactive anthocyanins and AvailOm^®^ further enhanced the vasculoprotective properties of these compounds, may offer new promising perspectives for preventing the onset of cardiovascular and cerebrovascular events.

## 1. Introduction

Cardio and cerebrovascular diseases including myocardial infarction (MI), coronary artery diseases (CAD) and stroke represent the leading cause of death worldwide and are tightly connected to endothelial dysfunction (ED), reduced nitric oxide (NO), and bioavailability, increased pro-oxidant factors, and altered vasomotor response.

Omega-3 (*ω*-3), a type of polyunsaturated essential fatty acid, has been reported as a necessary component for human health [1,2]. Numerous studies have revealed the beneficial effect of diets enriched in *ω*-3 polyunsaturated fatty acids (*ω*-3 PUFAs) in the prevention of cardiovascular diseases (CVD) [3,4]. The most common *ω*-3 PUFAs are α-linolenic acid C18:3 n-3, from vegetable sources, and eicosapentaenoic acid (EPA) and docosahexaenoic acid (DHA), found in fish oil. Although their potential beneficial effects include the reduced susceptibility to ventricular arrhythmia [5], delayed progression of the atherosclerotic plaque growth, anti-thrombogenic and antioxidant effect, anti-inflammatory effect, and mild hypotensive effect [6], the mechanisms by which they exert their cardiovascular protection have not been clarified. In addition, it is essential to remember that the high degree of unsaturation of *ω*-3 liquid oils makes them very susceptible to oxidation, which makes incorporation and absorption a significant challenge.

Similar beneficial action to that observed with *ω*-3 PUFAs, regards a different group of polyphenolic compounds present in fruits, vegetables, berries and red wine, the anthocyanins. It has been reported that anthocyanins possess both anti-inflammatory and anti-oxidative effects in vitro [7,8]. Animal studies conducted by Shaughnessy et al. [9] showed that 4 and 6 weeks of blueberry consumption in spontaneously hypertensive stroke-prone rats significantly lowered blood pressure. The dietary anthocyanin-rich bilberry extract also reduced blood glucose levels and enhanced insulin sensitivity in a mouse type 2 diabetes model [10]. Further, apolipoprotein E (ApoE)-deficient mice fed a diet rich in blueberries showed fewer atherosclerotic lesions and upregulation of antioxidant enzymes [11]. Some recent reviews summarized the beneficial effects of anthocyanins in human studies [12,13]. Daily supplementation of either 40, 80 or 320 mg of anthocyanins derived from bilberry and blackcurrant significantly attenuated serum inflammatory markers in a dose-dependent manner in patients with dyslipidemia [14]. Similar favorable effects on lipid profile were also observed after strawberry consumption. In a study conducted in healthy subjects, Zhang et al. [15] demonstrated that along with the reduction of low-density lipoproteins cholesterol (LDL-C) and triglycerides levels, 1 month of strawberry supplementation reduced serum levels of malondialdehyde, and isoprostanes. Furthermore, black raspberry extract administration reduced serum total cholesterol levels and inflammatory cytokines, thereby ameliorating vascular endothelial function in patients with metabolic syndrome during a 12-week follow-up [16].

However, despite a large body of evidence suggesting several health-promoting effects of anthocyanins, the variable concentration of other bioactive molecules present in these compounds made it nearly impossible to specifically attribute any observed beneficial effects to anthocyanins *per se*. Moreover, although individually, anthocyanins and *ω*-3 fatty acids are shown to exert a protective action on vascular function, the effect of the combination of these compounds on vasculature remains unexplored.

Thus, the present research was undertaken to investigate the impact and the underlying mechanism of a novel solid high-load *ω*-3 lysine complex formulation alone, or in combination with anthocyanins on vasorelaxation and protection of mice resistance arteries against oxidative stress induced by oxidized-LDL.

## 2. Material and Methods 

### 2.1. Reagents 

AvailOm^®^ and single *ω*-3 Fatty Acid (*ω*-3 FA), *ω*-3 Ethyl Ester (*ω*-3 EE), L-Lysin or Cyanidin-3-*O*-galactoside (C3-gal) and Delphinidin-3-o-arabinoside (DP3-ara) were obtained from EVONIK Industries (Essen, Germany). The oxLDL was acquired from Thermo Fisher Scientific (Waltham, MA, USA). All the inhibitors, powders, and solvents were purchased from Sigma-Aldrich, Merck, (St. Louis, MO, USA), unless otherwise specified.

### 2.2. Experimental Animals

All experiments involving animals were carried out according to the *Guide for the Care and Use of Laboratory Animals* published by the U.S. National Institutes of Health (NIH Publication No. 85-23, revised 2011) and were approved by the review board. Wild-type C57BL/6 mice (weighing ~25 g) (Jackson Laboratories, Bar Harbor, ME, USA) were used to perform vascular reactivity and molecular studies.

### 2.3. Vascular Reactivity Studies 

The vascular reactivity studies were performed on second-order branches of the mesenteric arterial tree collected from mice. In particular, vessels were mounted in a wire (DMT 210; Danish Myo Technology, Ann Arbor, MI, USA) or pressure myograph system (DMT 110P; Danish Myo Technology, Ann Arbor, MI, USA) containing Krebs solution (pH 7.4 at 37 °C in oxygenated 95% O_2_/5% CO_2_). 

We used 80 mmol/L of KCl to evaluate the vasoconstrictive response at the basal level. Phenylephrine (from 10^−9^ M to 10^−6^ M) was used to reproduce the 80% of maximal contraction. Acetylcholine (from 10^−9^ M to 10^−6^ M) or nitroglycerine (from 10^−9^ M to 10^−6^ M) was used to evaluate endothelial and smooth muscle-dependent vascular responses. The functional integrity of vessels was assessed by relaxation responses to acetylcholine (from 10^−9^ M to 10^−6^ M). 

Vascular responses were tested by administering increasing doses of AvailOm^®^ or single components such as L-Lysine, *ω*-3 Fatty Acid (*ω*-3 FA), *ω*-3 Ethyl Ester (*ω*-3 EE) form. In a different experimental setting, vascular studies were performed by administering Cyanidin-3-O-galactoside (C3-gal) or Delphinidin-3-o-arabinoside (DP3-ara) alone, or in combination with AvailOm^®^. Some experiments were performed in the presence of selective inhibitors of intracellular pathways, such as phosphatidylinositol-4,5-bisphosphate 3-kinase inhibitor (wortmannin, 10 µM, 1 h), the NOS inhibitor N-*ω*-nitro-l-arginine methyl ester (L-NAME, 300 µM, 30 min) or an inhibitor of AMPK, dorsomorphin (10 µM, 1 h) before data for dose-response curves were obtained. Mesenteric arteries were treated with AvailOm^®^ (100 µg/mL) or acetylcholine (10^−6^ M) artery within the last 30 min of 4-amino-5-methylamino-2,7-difluorofluorescein diacetate (DAF-FM), with or without L-NAME (300 μmol/L, 30 min). Cryosection of mesenteric arteries (5 µm thick), were observed under a fluorescence microscope and counterstained with hematoxylin and eosin.

### 2.4. Evaluation of ROS Production

Reactive oxygen species (ROS) production was evaluated by dihydroethidium staining (DHE, Life Technologies, Carlsbad, CA, USA). In detail, after incubation of 20 min with 5 µM of DHE, mesenteric arteries were observed and acquired under a fluorescence microscope (Zeiss Axiophot 2; Carl Zeiss Microscopy, Jena, Germany). 

Consequently, total ROS production was quantified with the membrane-permeable fluorescent probe, an analogous of 2,7-Dichlorodihydrofluorescein (DCDHF), Dihydrorhodamine 123 (DHR123) (Invitrogen, Thermo Fischer Scientific, Waltham, MA, USA) as previously described [17].

### 2.5. Statistical Analysis 

Data are presented as mean ± SEM. Statistical analysis was performed by two-way ANOVA followed by the Bonferroni post hoc test. Repeated measurements were analyzed by two-way ANOVA followed by the Bonferroni post hoc test. Differences were considered to be statistically significant when *p* < 0.05.

## 3. Results

### 3.1. AvailOm^®^ Evokes a Direct Vasorelaxant Action on Mice Mesenteric Arteries

**Figure 1:**
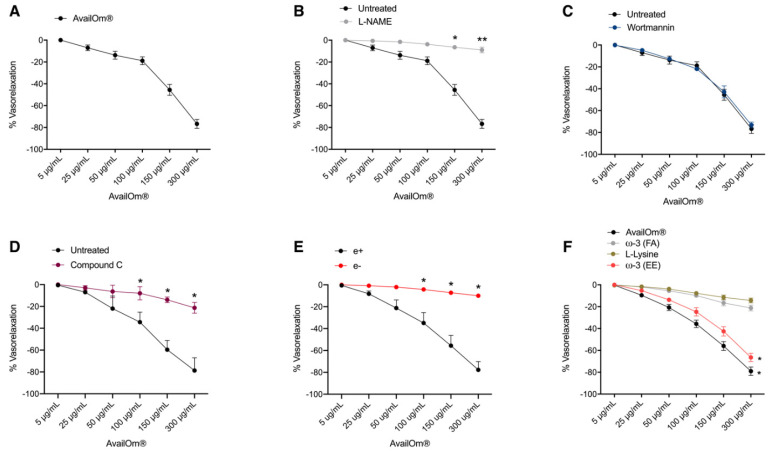
Evaluation of the vasorelaxant effects of AvailOm^®^ and its fractions in mice mesenteric arteries. (**A**–**D**) Vascular reactivity function of phenylephrine-precontracted mice vessels to increasing doses of AvailOm^®^ (5–300 µg/mL) (N = 5). (**B**) Vascular response of phenylephrine-precontracted mice mesenteric arteries to increasing doses of AvailOm^®^ in in the presence of L-NAME, (**C**) wortmannin, (**D**) Compound C or (**E**) in vessels with endothelium (e+) or without endothelium (e−). (**F**) Comparison of the vasorelaxant effect of AvailOm^®^, *ω*-3 FA, *ω*-3 EE or L-Lysine. Statistical analyses were performed using two-way ANOVA followed by Bonferroni post hoc test. * *p* < 0.05; ** *p* < 0.01.

Alteration of the vascular response of resistance arteries is a determining factor in the development of cardiovascular complications. The potential direct vascular action of AvailOm^®^ was assessed by performing vascular reactivity studies on pre-constricted mice mesenteric arteries exposed to increasing doses of AvailOm^®^ (5–300 µg/mL). AvailOm^®^ exerted a direct dose-dependent vasorelaxant action (Figure 1A). Since the inhibition of the eNOS enzyme, by N-*ω*-nitro-l-arginine methyl ester (L-NAME), completely abolished relaxing responses to AvailOm^®^, its effect is due to the stimulation of nitric oxide production (Figure 1B). Being one of the major enzymes involved in eNOS activation, subsequent studies were performed in the presence of a phosphoinositide 3-kinase (PI3K) inhibitor, demonstrating that this mechanism is not involved in the direct vascular action exerted by AvailOm^®^ (Figure 1C). Interestingly, in the presence of a selective AMPK inhibitor, Compound C, AvailOm^®^ completely lost its capability to evoke endothelial-dependent vasorelaxation (Figure 1D). Vasorelaxation promoted by AvailOm^®^ was completely abolished after endothelium removal, demonstrating that endothelium represents the main target of the compound (Figure 1E).

Since AvailOm^®^ contains a high-load *ω*-3 (FA & EE) lysine complex, subsequent studies were performed to identify the specific component able to evoke the vasorelaxant effect with a strong efficacy. While assessment of vascular response to L-Lysine or *ω*-3 FA did not show any vasorelaxant effect (Figure 1F), *ω*-3 EE was able to induce a dose-dependent vasorelaxation of mesenteric arteries with similar efficacy observed by AvailOm^®^ alone (Figure 1F), thus suggesting that the direct action on vascular tone is due to the presence of EE in the compound.

### 3.2. AvailOm^®^ Prevents Vascular Oxidative Stress Damage Induced by oxLDL 

**Figure 2:**
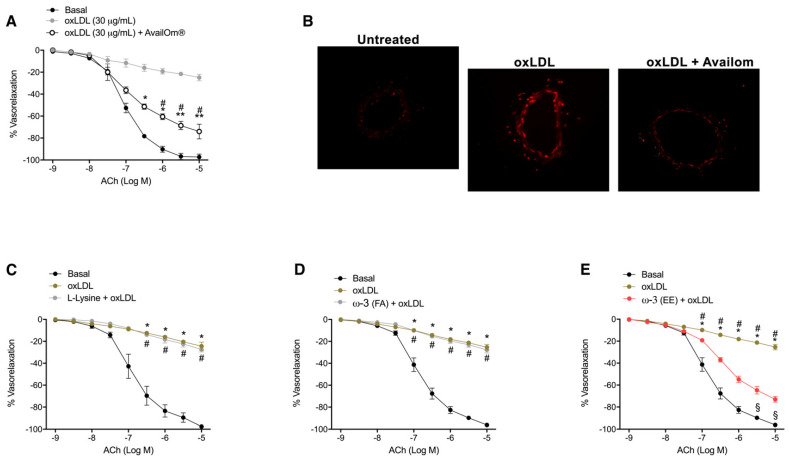
Vasculoprotective effect of *ω*-3 Ethyl Ester against oxLDL-induced oxidative stress and endothelial dysfunction. (**A**) Vascular reactivity studies of phenylephrine-precontracted mice mesenteric arteries exposed to increasing doses of ACh (10^−9^ to M 10^−5^ M) after exposure to oxLDL for 30 min and to 1 h to AvailOm^®^ (100 µg/mL). (**B**) Representative high-power micrographs of mice mesenteric arteries cryosections loaded with dihydroethidium probe at the concentration of 5 μM. Vessels were pre-treated with a single compound (100 µg/mL) for 1 h and then stimulated with oxLDL for 30 min before the acquisition. (**C**–**E**) Vascular response of phenylephrine-precontracted mice mesenteric arteries to increasing doses of ACh (10^−9^ to M 10^−5^ M) after exposure to oxLDL for 30 min and to 1 h to L-Lysine, *ω*-3-FA or *ω*-3 EE (100 µg/mL). Statistical analyses were performed using two-way ANOVA followed by Bonferroni post hoc test. * *p* < 0.05; ** *p* < 0.01; # *p* < 0.05; § *p <* 0.05.

**Figure 3:**
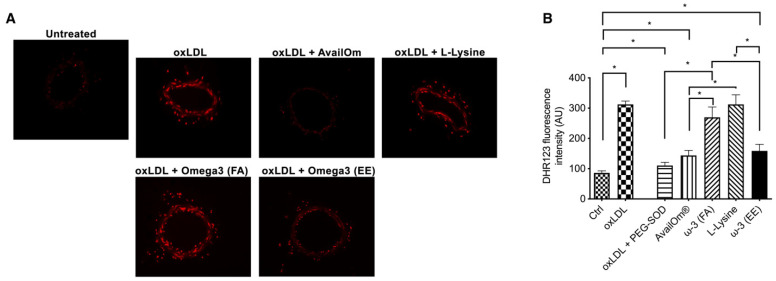
Effect of AvailOm^®^ in vascular oxidative stress and endothelial dysfunction induced by oxLDL. (**A**) Representative high-power micrographs of mice mesenteric arteries cryosections loaded with 5 μM of dihydroethidium dye. Mesenteric arteries were pre-treated with a single compound (100 µg/mL) for 1 h and then stimulated with oxLDL for 30 min before the acquisition. (**B**) Measurement of ROS production by DHR123 in vessels treated with single compounds. AU, arbitrary units. Statistical analyses were performed using one-way ANOVA followed by Bonferroni post hoc test. * *p* < 0.05.

Subsequently, the potential vasculoprotective effect of AvailOm^®^ was also investigated under conditions of increased oxidative stress. Pre-treatment with AvailOm^®^ (100 µg/mL) of mesenteric arteries revealed significant protection from oxidized low-density lipoprotein (oxLDL)-induced endothelial dysfunction of mice mesenteric arteries, as demonstrated by dose-response curves to acetylcholine (Ach) (Figure 2A). As assessed by dihydroethidium (DHE), AvailOm^®^ also attenuated oxLDL-evoked oxidative stress in vessels (Figure 2B). Interestingly, the protection assessment from oxLDL-evoked vascular impairment revealed the EE form of *ω*-3 as the key component that owns the beneficial cardiovascular properties of AvailOm^®^ (Figure 2C–E). Finally, the qualitative and quantitative assessment of oxidative stress by DHE and DHR123, demonstrated that *ω*-3 EE reproduced the same effect of AvailOm^®^ alone, supporting the notion that EE is the most effective biochemical form able to protect from oxLDL-evoked vascular oxidative stress in ex vivo treated-mesenteric arteries (Figure 3A,B). 

### 3.3. AvailOm^®^ in Combination with the Most Potent Anthocyanins, Exerts the Highest Vasorelaxant Effect

**Figure 4:**
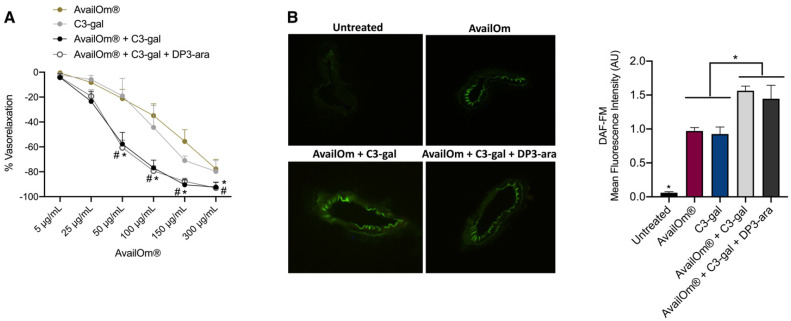
Effect of AvailOm^®^ in combination with different anthocyanins on vascular function and nitric oxide production. (**A**) Vascular response of phenylephrine-precontracted mice vessels to increasing doses of AvailOm^®^ (5–150 µg/mL) or to C3-gal, or to AvalOm^®^ plus C3-gal or AvalOm^®^ plus C3-gal plus DP3-ara with a ratio ½:½ or 1/3 respectively (N = 3). (**B**) Representative high-power micrographs of mice mesenteric arteries cryosections loaded for 2 h with 4,5-diaminofluorescein (DAF-FM) after treatment with AvailOm^®^ or single combination bar graph shows the mean fluorescence intensity of section for each compound (N = 4). * *p* < 0.05 AvailOm^®^ + C3-gal vs. AvailOm^®^ alone; # *p* < 0.05 AvailOm^®^ + C3-gal + DP3-ara vs. AvailOm^®^ alone.

In a previous study, Cyanidin-3-O-galactoside (C3-gal) and C3-gal plus Delphinidin-3-o-arabinoside (DP3-ara) represent the most potent anthocyanins able to exert vasorelaxant effects [17]. Thus, the potential vascular impact of AvailOm^®^ was also assessed in combination with them in a ratio of ½:½. In the presence of C3-gal or C3-gal plus DP3-ara, AvailOm^®^ exerted an even more powerful vasorelaxant effect, showing a significant improvement of endothelial-dependent vasorelaxation at 50, 100, 150, and 300 μg/mL as compared to AvailOm^®^ alone (Figure 4A). In addition, the assessment of nitric oxide production by DAF-FM revealed that AvailOm^®^ in the presence of C3-gal or C3-gal plus DP3-ara with a 1:1 or 1:1:1 ratio significantly increased NO production compared to AvailOm^®^ at 100 μg/mL (Figure 4B). 

**Figure 5:**
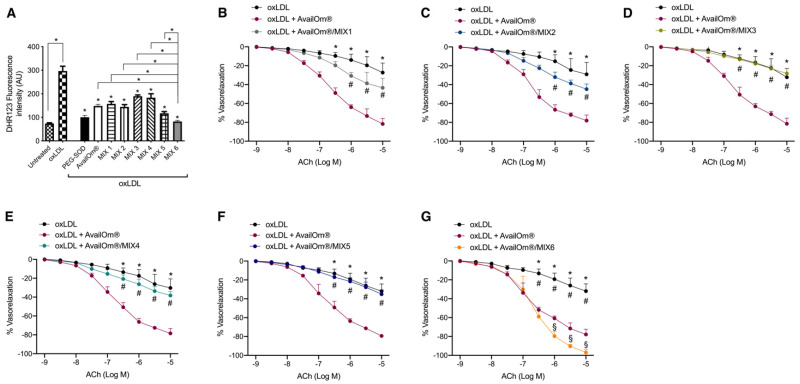
Vasculoprotective effects of the combination of AvailOm^®^ and different anthocyanins’ MIX against oxLDL-mediated ROS overproduction. (**A**) Measurement of ROS production by DHR123 in vessels treated with oxLDL alone or with PEG-SOD, AvailOm^®^, or AvailOm^®^ plus MIX 1: C3-glu + C3-gal; MIX 2: Mal3-glu + Mal3-gal; MIX 3: C3-glu + DP3-glu + Mal3-glu; MIX 4: Mal3-gal + PEO3-gal; MIX 5: C3-glu + DP3-glu + C3-rut + Mal3-glu + Mal3-gal + PEO3-gal or MIX6: C3-glu + DP3-glu + Mal3-glu + Mal3-gal + PEO3-gal. Statistical analyses were performed using one-way ANOVA followed by Bonferroni post hoc test. *p < 0.05. (**B**–**G**) Vascular response of phenylephrine-precontracted mice mesenteric arteries to increasing doses of ACh (10^−9^ to M 10^−5^ M) after exposure to oxLDL for 30 min and then to AvailOm^®^ alone or AvailOm^®^ in combination with MIX1, MIX2, MIX3, MIX4, MIX5 or MIX6. * *p* < 0.05 vs. oxLDL + AvailOm^®^. # *p* < 0.05 oxLDL + AvailOm^®^; § *p* < 0.05 vs. oxLDL + AvailOm^®^.

The effect of AvailOm^®^ on ROS production was also investigated in combination with different anthocyanins’ MIX. First of all, AvailOm^®^ plus MIX6 (C3-glu + DP3-glu + Mal3-glu + Mal3-gal + PEO3-gal), respecting a ratio of 1:6 of each product, provided greater protection against oxLDL-evoked oxidative stress than AvailOm^®^ alone or AvailOm^®^ in combination with MIX 1 (C3-glu + C3-gal), MIX 2 (Mal3-glu + Mal3-gal), MIX 3 (C3-glu + DP3-glu + Mal3-glu), MIX 4 (Mal3-gal + PEO3-gal) or MIX 5: C3-glu + DP3-glu + C3-rut + Mal3-glu + Mal3-gal + PEO3-gal (Figure 5A). Consistent with these results, the combination of AvailOm^®^ and MIX6 turned out to be more effective than AvailOm^®^ alone or in combination with the other anthocyanins’ MIX in preventing oxLDL-induced endothelial dysfunction mesenteric arteries (Figure 5B–G).

## 4. Discussion

Starting from the first cross-cultural epidemiological studies in the 1970s, [18], the body of evidence supporting the role of *ω*-3 in the prevention of cardiovascular disease (CVD), has continued to increase. Many studies have shown enhanced endothelium-dependent vasodilation after *ω*-3 supplementation in patients with type 2 diabetes [19] and hyperlipidemia [20].

However, it is known that *ω*-3 oils are prone to oxidative deterioration and the development of unpleasant odors and flavors even when precautions such as protection from light and oxygen and storage at low temperatures are taken. Oxidation that affects the nutritional quality and safety of the oil for consumers has been the factor that has limited its use in foods. In addition, when *ω*-3 fatty acids are consumed as triglycerides or ethyl esters, they must be hydrolyzed to the free fatty acid by gastric enzymes. Recently, a novel high-load *ω*-3 lysine complex formulation, AvailOm^®^, has been developed. It consists of a pre-converted lysine complex that rapidly dissociates to the free fatty acid, absorbing it immediately, thus overcoming the pre-conversion step and ensuring higher *ω*-3 stability. However, what vascular effects were evoked and what molecular mechanisms were recruited by this new solid-phase formulation of *ω*-3 were still unknown.

This study showed that AvailOm^®^ produces a direct dose-dependent vasorelaxation via the AMPK/eNOS-mediated signaling axis through vascular reactivity studies and molecular analysis. Moreover, the *ω*-3 EE form was identified as the main component able to exert direct vasorelaxant and antioxidant effects to the same extent as AvailOm^®^. 

Numerous primary and secondary prevention epidemiological studies suggested that dietary intake of *ω*-3, including the two primary compounds eicosapentaenoic acid (EPA) and docosahexaenoic acid (DHA), can reduce the risk of cardiovascular diseases [21,22]. Moreover, dietary supplementation with *ω*-3 PUFA-rich fish or fish oil significantly reduced blood pressure levels in hypertensive patients and improved endothelium-dependent vasorelaxation in patients with coronary artery disease and type 2 diabetes [23]. 

This study showed that AvailOm^®^ evoked a dose-dependent vasorelaxation mediated by eNOS activation driven by an essential intracellular enzyme, 5′ AMP-activated protein kinase (AMPK), a crucial molecule that hinders or delays the process of fibrogenesis, protecting various organs and tissues, including heart, kidney, liver, lung [24]. Moreover, AMPK contributes to the anti-aging action of FGF21, contributing to the reduction of cerebrovascular aging-related diseases, such as stroke and neurodegenerative diseases [25]. Increasing doses of AvailOm^®^ ultimately failed to induce endothelial-dependent vasorelaxation in the presence of Compound C, which selectively inhibited AMPK activation. 

Several studies suggest that oxLDL exerts an essential role in the pathogenesis of atherosclerosis. Its abundance in atherosclerotic vascular lesions also supports this concept. The endothelial layer represents a major target for oxLDL-mediated vascular injury in atherogenesis and plays a fundamental role in regulating vascular homeostasis by modulating vasomotor tone. Thus, altered endothelial function is considered to be an early pathogenic event able to predict cardio and cerebrovascular diseases [26,27]. 

The possible beneficial effects of AvailOm^®^ on vascular function were also tested in an ex vivo model of vascular dysfunction and oxidative stress evoked by oxidized low-density lipoprotein. AvailOm^®^ effectively protected mice mesenteric arteries from oxLDL-evoked vascular oxidative stress and endothelial dysfunction. Moreover, the specific analysis revealed that between the components of AvailOm^®^, while L-Lysine and *ω*-3-Fatty acid did not evoke any effects on endothelial protection, *ω*-3-Ethyl ester represented the only fraction of AvailOm^®^ able to produce vascular protection, by completely restoring the ACh-mediated vasorelaxation. Oxidative stress analysis revealed that *ω*-3-Ethyl ester significantly counteracts the oxLDL-evoked oxidative stress. 

Anthocyanins from blueberries or red wine have been shown their capability to improve endothelium-dependent vasorelaxation in experimental models [28]. In the past decades, several clinical studies have hinted at the positive role of anthocyanins in preserving cardiovascular health. Curtis and co-authors [29] demonstrated that higher intake of blueberries for 6 months was associated with a reduced systemic arterial stiffness, improved endothelial function, and decreased HDL cholesterol concentrations in patients with metabolic syndrome. Similarly, Rodriguez-Mateos et al. [30] performed a nutrigenomic study to explore the mechanism of action of anthocyanins in vivo. Daily 1-month blueberry consumption resulted in a sustained improvement in endothelial function and a 5 mmHg reduction in 24-h systolic blood pressure [30], a magnitude similarly to that commonly observed in clinical studies using antihypertensive drugs (e.g., ACE inhibitors) [31]. Additionally, the authors showed that the endothelial function improvement was directly correlated with plasma anthocyanins metabolites.

To date, the potential beneficial vascular effects of *ω*-3-Fatty acid in combination with specific anthocyanins have not yet been investigated. Using an innovative experimental approach, this study demonstrated that the concomitant treatment of AvailOm^®^ with the most potent anthocyanins further enhanced its vasorelaxant action.

Interestingly, the MIX AvailOm^®^ with C3-gal (1/2:1/2) or with C-3gal plus DP3-ara (1/3:1/3:1/3) resulted in a significant improvement of dose-responses vasorelaxant effects in association with both C3-gal and C3-gal plus DP3-ara, which represent the most effective vasorelaxant anthocyanins. 

To investigate the vascular action of the AvailOm^®^ in combination with anthocyanins on the cardiovascular protection, different anthocyanins MIXs with AvailOm^®^ were tested on acetylcholine-evoked endothelial vasorelaxation in mesenteric arteries exposed to oxLDL. Results showed that the combination of AvailOm^®^ with MiX6, which contains C3-glu + DP3-glu + Mal3-glu + Mal3-gal + PEO3-gal, maintaining a 1:6 ratio, was able to improve endothelial-dependent vasorelaxation and reduce the oxidative stress after oxLDL treatment. Although this is the first study investigating the possible vascular effects of a novel solid high-load *ω*-3 lysine complex formulation alone, or in combination with powerful anthocyanins, the limitation of the study is that the data obtained came from studies conducted in ex vivo murine arteries, thus limiting the direct translatability of the results. Therefore, further studies in both large animal models and humans are needed to translate the results obtained to human pathophysiology.

## 5. Conclusions

Overall, the present study showed, for the first time, that a novel high-load *ω*-3 lysine complex formulation (AvailOm^®^) exerts a direct vasorelaxant effect in ex vivo treated mesenteric arteries by modulating AMPK1, favoring eNOS stimulation and nitric oxide production. AvailOm^®^ effectively inhibited the endothelial dysfunction and vascular oxidative stress caused by oxLDL. Moreover, there is potential for AvailOm^®^ to be used in combination with specific anthocyanins since these compounds induce enhanced vascular protection at lower doses. Therefore, these data open a new scenario for developing a novel compound composed of *ω*-3-Ethyl esters combined with anthocyanins to obtain maximum vascular prevention. 

## Data Availability

The data presented in this study are available on request from the corresponding author. The data are not publicly available due to the development of patent application.
